# Supporting Physical Activity in the Childcare Environment (SPACE): rationale and study protocol for a cluster randomized controlled trial

**DOI:** 10.1186/s12889-016-2775-9

**Published:** 2016-02-03

**Authors:** Patricia Tucker, Shauna M. Burke, Anca Gaston, Jennifer D. Irwin, Andrew M. Johnson, Brian W. Timmons, Leigh M. Vanderloo, Molly Driediger

**Affiliations:** 1 School of Occupational Therapy, Faculty of Health Sciences, University of Western Ontario, 1201 Western Road, Elborn College, Room 2547, London, ON N6G 1H1 Canada; 2 School of Health Studies, Faculty of Health Sciences, University of Western Ontario, London, Ontario Canada; 3 School of Kinesiology, Faculty of Health Sciences, University of Western Ontario, London, Ontario Canada; 4 Department of Pediatrics, McMaster University, Hamilton, Ontario Canada; 5 Health and Rehabilitation Sciences, Faculty of Health Sciences, University of Western Ontario, London, Ontario Canada

**Keywords:** Physical activity, Childcare, Preschool, Sedentary behaviour, Outdoor playtime, Early years, Intervention, Protocol

## Abstract

**Background:**

Young children are prone to low levels of physical activity in childcare. Researchers have identified that preschoolers tend to be more active outdoors than indoors, with higher activity levels occurring during the first 10 minutes of outdoor playtime. Additionally, interventions incorporating either staff training or the inclusion of play equipment have been effective at increasing children’s activity in this setting. As such, the overarching objective of the *Supporting Physical Activity in the Childcare Environment* (SPACE) intervention is to improve the physical activity levels of preschoolers during childcare hours, utilizing a combination of the above components*.* The purpose of this manuscript is to provide a detailed account of the protocol, innovative methods, and evaluation plans used in the implementation of the SPACE study; in an effort to support the development of further research in this field.

**Methods/Design:**

The SPACE study, a cluster randomized controlled trial, involves 22 childcare centres randomly allocated to either the experimental (*n* = 11) or the control (*n* = 11) group. Childcare centres receiving the intervention will adopt an 8-week physical activity intervention with the following components: 1. shorter, more frequent bouts of outdoor playtime (4 × 30 min periods rather than 2 × 60 min periods); 2. new portable play equipment (e.g., obstacle course, balls); and, 3. staff training (1 × 4 hr workshop). Actical accelerometers will be used to assess total physical activity with measurements taken at baseline (i.e., week 0), immediately post-intervention (i.e., week 8), and at 6- and 12-month follow-up. As secondary objectives, we aim to evaluate the effectiveness of the intervention on preschoolers’: a) sedentary time; b) standardized body mass index scores (percentiles); c) health-related quality of life; and childcare providers’ physical activity-related knowledge and self-efficacy to implement physical activity.

**Discussion:**

The SPACE study aims to increase the low levels of physical activity observed within childcare centres. The findings of this work may be useful to policy makers and childcare providers to consider modifications to the current childcare curriculum and associated outdoor play time.

**Trial registration:**

ISRCTN70604107 (October 8, 2014)

## Background

Preschoolers’ (2.5-5 years) physical activity levels have received extensive attention in recent years [1–8]. In a systematic review of the literature, Tucker found that only 54 % of preschoolers from 7 countries engaged in at least 60 minutes of daily activity [9]. Studies have also shown that physical activity levels tend to decrease with age [10], with a 50 % reduction between the ages of 3 and 4 years, and statistically significantly lower again by age 5 [11]. This is disconcerting as Canada’s Physical Activity Guidelines for the Early Years [12] recommend that preschoolers should engage in 180 minutes of daily activity (at any intensity). Thus, early intervention is crucial given that physical activity plays a pivotal role in children’s overall health and is associated with many positive health outcomes, including improved weight status, cardiovascular health, psychosocial and cognitive development, and bone health [13]. Sedentary behaviour, also noted to be prevalent among the preschool population [14], has been identified as detrimental to preschoolers’ health [15].

A growing body of literature, both Canadian and international, [1, 2, 16, 17] has been undertaken exploring physical activity levels among preschoolers in childcare centres, and many researchers have reported that their activity levels are dismally low. Specific to this environment, Temple and colleagues [16] and Vanderloo et al. [2] found that preschoolers were well below the guidelines averaging 148 and 133 minutes of physical activity, respectively. When exploring moderate-to-vigorous physical activity (MVPA) specifically, researchers have provided evidence to suggest that preschoolers engaged in only 1.54 and 1.76 min/day while in care [2, 16]. In combination with these low levels of physical activity participation, preschoolers’ have been noted to partake in upwards of 42 min/hr of sedentary time within the childcare setting [14]. These trends are alarming, particularly because while opportunity exists outside of childcare for kids to be active, parents have acknowledge they rely on childcare facilities to ensure their kids are moving [18].

Over 80 % of Canadian preschoolers attend some form of childcare or early childhood education program [19]. Given that children learn many lessons while in out-of-home care, including those related to nutrition, screen-viewing, and physical activity [20], researchers have purported that policies and programming in early learning environments may serve as ideal avenues for increasing physical activity and enhancing the overall health of this population [21]. In fact, researchers have found that the childcare centre accounts for approximately 50 % of the variation in preschoolers’ physical activity [17, 22]. Furthermore, a recent Canadian study found a positive correlation between the number of hours preschoolers spend in childcare and the prevalence of obesity, and that those enrolled in centre-based childcare had higher rates of obesity than those cared for by a parent or relative [23]. In light of these findings, combined with the disturbingly low rates of physical activity accumulated in Canadian childcare centres [1, 2, 16], evidence-based efforts to combat these trends are essential.

Recently, a meta-analysis [24] and two systematic reviews [25, 26] were undertaken to explore characteristics and efficacy of interventions aimed at preschoolers. Gordon and colleagues [24] explored the effectiveness of physical activity interventions (*n* = 15) and found that programs offered in the childcare facility, led by teachers (rather than parents), and which incorporated unstructured playtime and offered outdoor activity, were most effective. These authors reported that physical activity interventions had a small-to-moderate effect on overall physical activity (Hedges *g* = 0.44; *p* < .05) and a moderate effect on MVPA (Hedges *g* = 0.51; *p* < .05). When examining interventions in the childcare setting only, Ward and colleagues (*n* = 9) concluded that the majority of interventions were effective at increasing activity levels of participating preschoolers, and they identified organized physical activity sessions integrated into the curriculum, appropriate physical activity staff training, and portable equipment, as important characteristics [26]. Finally, Hinkley et al. completed a review to explore the correlates of physical activity participation among preschoolers and reported that “boys were more active than girls…and that children who spent more time outdoors were more active than children who spent less time outdoors” (p.435) [25]. Based on this evidence, it is clear that providing portable play equipment (e.g., balls, tricycles, hula hoops), staff training, and access to outdoor playtime is important for facilitating physical activity participation among preschool-aged children. Based on these above studies, and other Canadian research efforts [27], it is clear that outdoor playtime is instrumental for improving preschoolers’ physical activity in childcare. Previous work has reported that young children are most active during their first 10 minutes outdoors [28]; consequently, it is possible that the more opportunities for outdoor play preschoolers are provided in childcare, the more minutes of physical activity they are likely to accumulate.

### Study rationale

A consolidated understanding of the effective characteristics of physical activity interventions targeting the preschool population is now available, and this information, combined with recent evidence which highlights the importance of outdoor playtime for preschoolers, serves as a call for effective action. While researchers are striving to improve the activity levels of preschoolers in childcare at an international level, we are only aware of one other childcare physical activity intervention undertaken in Canada [29], which adopted a staff training focus.

### Study objectives

The primary objective of the SPACE study is to implement and evaluate the effectiveness of an evidence-based intervention at increasing total physical activity (TPA) among preschoolers enrolled in centre-based childcare. As secondary objectives, we aim to evaluate the effectiveness of the intervention with regard to: a) decreasing sedentary time; b) decreasing preschoolers’ body mass index scores (percentiles); c) increasing preschoolers’ health-related quality of life; d) increasing childcare providers’ physical activity-related knowledge; e) increasing childcare providers’ self-efficacy to implement physical activity programming with the children in their care; and f) examining whether sex or child temperament influences preschoolers’ physical activity levels and sedentary time.

### Hypotheses

We hypothesize that preschoolers in the experimental group (i.e., receiving the intervention) would display increased rates of TPA (min/hr) from baseline to post-intervention, while no change would be observed for preschoolers in the control condition. While levels of TPA are expected to decrease at 6- and 12-month follow-up, we believe that activity levels at these time points will still be higher than those recorded at baseline for the experimental group. We also anticipate decreased sedentary time and improvements in health-related quality of life post-intervention among preschoolers in the intervention group compared with the control group. Given the natural growth that occurs among children within this age range, some change in BMI is expected among children in both groups, although we would not anticipate any change in standardized BMI scores (i.e., percentiles) for children in the control group. For childcare providers from the experimental group, we expect an increase in physical activity-related knowledge and self-efficacy for implementing physical activity programming with preschoolers in their care. Furthermore, we anticipate that male preschoolers will accrue higher levels of TPA (and lower sedentary time) in comparison to their female counterparts. Although minimal evidence on the impact of child temperament on physical activity levels exists, we expect that child temperament may be a contributing factor to preschoolers’ physical activity levels.

## Methods

### Trial design

Following the PRECEDE-PROCEED model for health promotion program planning [30], and in line with the Consolidated Standards of Reporting Trials (CONSORT) statement [31], a cluster randomized controlled trial, involving the implementation and evaluation (i.e., process, impact, and outcome evaluation) of the multi-faceted SPACE study will be undertaken. Due to the nature of the intervention, a double-blind study design is not possible, but to maintain the rigor of the study, a single-blinded design will be adopted (all assessments will be conducted by research staff who remain unaware of group assignment). This study received ethical approval from the University of Western Ontario's Research Ethics Board (REB# 105779) and has been assigned an International Standard Randomised Controlled Trial Number (ISRCTN70604107).

### Sample size

A recent meta-analysis exploring the effectiveness of 15 physical activity interventions targeting preschoolers by Gordon et al. [24] reported that most studies had a small-to-moderate effect (Hedges *g* = 0.44) for change in this population’s TPA levels after participating in an intervention. According to Cohen [32], a two-group design would thus require 83 participants per group assuming a small-to-moderate effect, a power level of .80, and an alpha of .05. Childcare centres will be targeted as units (clusters); therefore, the sample size will be adjusted to account for the clustering effect, where:

$$ \mathrm{D}=1+\left(\mathrm{k}\hbox{-} 1\right)\uprho =1+\left(16\hbox{-} 1\right)(0.05)=1.75 $$

(**D** = design effect; ***k*** = anticipated cluster size [class size in this case]; ρ = the intra-cluster correlation coefficient, a measure of the degree of homogeneity among cluster subjects for a particular outcome investigated). Therefore, the design effect for an average cluster size of 16 children was 1 + 0.05(16-1) = 1.75. Thus, the sample size of each group will be inflated to 83 × 1.75 = 145. We anticipate a loss to follow-up rate of approximately 20 % (a rate comparable with other interventions with this population [33, 34], our goal is to recruit a minimum of 174 preschoolers per group. Therefore, the final targeted sample size is 348 preschoolers; we anticipate needing participation from 22 childcare centres to reach this participant pool.

### Recruitment and randomization

**Fig. 1:**
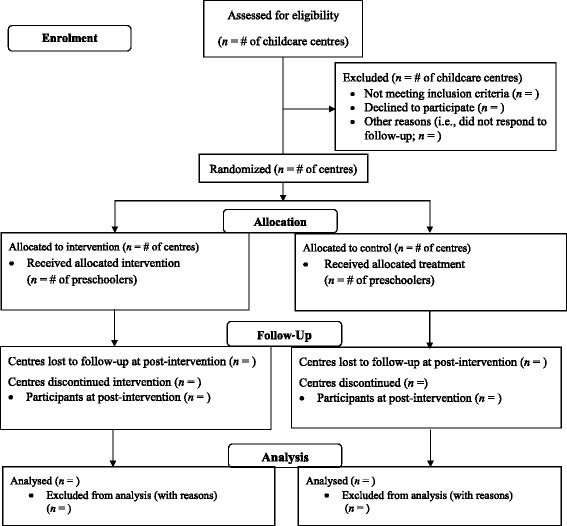
CONSORT flow diagram for the SPACE study

Childcare centres will be randomly selected from a list of 69 facilities using a database search on the Ontario Ministry of Education website [35]. The directors at randomly selected childcare centres will be contacted by phone by the project coordinator to explain the nature of the study and to invite participation. Once verbal consent has been received from 22 childcare directors, the centres will be randomly assigned to the experimental or control condition by the project coordinator. It would be logistically impossible to assign individual participants within the same childcare centre to the experimental and control groups; as such, the childcare centre will be the unit of randomization. Research Randomizer (www.randomizer.org) will be used to allocate the centres into two equal groups of 11 using a blocked design (with an allocation ratio of 1:1). This website will be used to generate a number (either 0 or 1) and based on the number produced, the centre will be assigned to either the control (i.e., “0”) or the experimental (i.e., “1”) group. The project coordinator will conceal the assignment of clusters that received the intervention (i.e., experimental) and those that continued their normal routine (i.e., control) from other research personnel. Random allocation of participating centres will occur prior to individual participant recruitment (Fig. 1).

### Inclusion/exclusion criteria

#### Childcare centres

Centre-based childcare facilities in London, Canada with at least one preschool classroom, where the staff and children are English-speaking and the educators are willing to participate, will be eligible to participate in this study.

#### Preschoolers

Children between the ages of 2.5-4 years who are enrolled in a preschool classroom of an enrolled childcare centre will be eligible to participate. The eligible child’s English-speaking parent/guardian will be required to provide consent prior to participation.

### Description of SPACE intervention

#### Experimental condition

The 8-week SPACE intervention will involve environmental modifications, staff training, and curriculum changes for the preschool classrooms in participating childcare facilities. First, *environmental modifications* will entail the addition of portable play equipment (for use indoors and outdoors) that has been shown to predict physical activity levels among preschoolers [36, 37]. The package will include items such as: balls, hula hoop activity pack, obstacle course, stepping domes, ribbons, a hopscotch play mat, and hop-along bouncers. The equipment will be delivered directly to each centre at the start of the 8-week intervention period. Staff will be asked to rotate the equipment available (e.g., offering different pieces each day) and to ensure it is used for the duration of the intervention. Childcare centres will keep all physical activity equipment at the conclusion of the intervention period. Second, *physical activity-related staff training* will involve one 4-hour session with childcare providers and directors. The principal investigator, the project coordinator, and a consultant who is experienced in delivering physical activity education for childcare personnel will lead the staff training. In this session, childcare staff will be provided with information regarding: (a) the Canadian physical activity and sedentary behaviour guidelines for preschoolers [38, 39]; (b) the need for shorter, more frequent bouts of outdoor activity [7, 28, 40, 41]; (c) how to incorporate physical activity into the childcare centres’ indoor curriculum; and, (d) overcoming physical activity barriers, like poor weather and limited resources*.* Third, *curriculum modifications* will target both indoor and outdoor play periods provided to participating preschoolers. Specifically, preschoolers in the experimental condition will receive four 30-minute outdoor play periods per day rather than two 60-minute outdoor play periods per day (which is the current practice in Ontario). This re-structured outdoor time is designed as free play, in which the preschoolers could interact with the new portable play equipment. The modified outdoor playtime arrangement will last the full 8 week duration of the intervention. Because childcare staff have previously requested “guest physical activity instructors” [42] the indoor curriculum modifications will include the use of a fitness instructor (who has experience leading physical activities for young children) who will provide one 30-minute exercise class for participating preschool classrooms during the intervention period [43]. This session will consist of indoor physical activities (e.g., dancing, follow the leader, fitness moves, etc.) that the staff could adopt within their programming.

In an effort to help maintain the newly acquired physical activity-based training and knowledge that the childcare providers will receive during the intervention, one booster session will be offered to each centre in the experimental group approximately 4 months post-intervention. These sessions will be led by either the project coordinator or the early year’s public health nurse, and will include reminders of the physical activity guidelines for this age group, creative ways to get young child moving, as well as resource sharing.

#### Control condition

Childcare centres randomly assigned to the control group will continue their typical daily curriculum and outdoor play sessions (i.e., two 60-minute unstructured outdoor play periods) for the duration of the intervention and follow-up periods. Upon completion of the SPACE study, all centres allocated to this group may opt to receive the intervention staff training.

### Tools of assessment

A variety of tools will be used to assess the impact of the SPACE intervention on preschoolers' physical activity and health-related outcomes (Table 1).

**Table 1:** Outcome assessments for the SPACE study

		Control Condition				Experimental Condition			
		Baseline (week 0)	Post-Int (week 8)	6-mo	12-mo	Baseline (week 0)	Post-Int (week 8)	6-mo	12-mo
Preschoolers^a^	Consent Form	x				x			
	Family Demographic Q	x				x			
	Objective Physical Activity and Sedentary Time (Actical data)	x	x	x	x	x	x	x	x
	Anthropometrics	x	x	x	x	x	x	x	x
	Children’s Behaviour Q	x	x	x	x	x	x	x	x
	PedsQL 4.0 Generic Core Scales Q	x	x	x	x	x	x	x	x
Childcare Staff	Consent Form	x				x			
	Demographic Q	x				x			
	Outdoor Playtime Logb	x	x			x	x		
	Self-Efficacy Q	x	x	x	x	x	x	x	x
	Physical Activity Knowledge Q (inclusive of IPAQ – SF)	x	x	x	x	x	x	x	x
	Program Evaluation Survey						x		
	Focus Groups						x		

*Note*. Post-Int = post-intervention; ^a^or completed by preschoolers’ parents/guardians, ^b^the outdoor playtime log was completed by childcare staff every week of the 8 week intervention

#### Primary outcome measures

##### Preschoolers’ physical activity levels

Preschoolers’ TPA (min/hr) during childcare hours will be objectively assessed using Actical™ accelerometers (MiniMitter, Bend, Oregon). Among the preschool population in particular, these small motion sensor devices have been shown to provide reliable and valid estimates of physical activity [31, 44]; they provide detailed and accurate information on the duration, frequency, intensity of activity (i.e., light, moderate, vigorous) as well as the dates/times at which these activities occur. Consistent with the preschool literature, and in an effort to ensure an accurate depiction of this group’s activity behaviours, an epoch length of 15 seconds will be applied [44]. Participating preschoolers will be asked to wear the accelerometers during childcare hours only for five consecutive days (i.e., Monday to Friday, or when in care) prior to the introduction of the intervention (i.e., baseline), for one week at the completion of the intervention period (i.e., week 8), and for one week at the 6- and 12-month follow-up periods. Accelerometers will be secured to the participants’ right hip (i.e., above the iliac crest) using an adjustable elastic belt and will be programmed to begin collecting activity data on the morning (i.e., 7 am) of the first day of data collection (i.e., Monday). At each of these data collection periods, childcare staff will be asked to keep a log of the times the devices are placed on the children and removed each day. Accelerometry training (i.e., how to place/remove the accelerometers, how to complete the wear time log) will be provided to the childcare staff by the research assistants prior to each data collection period. Minutes of sedentary time (≤24.75 counts⋅15 s^-1^) [45], light physical activity (LPA; between 25 and 287.25 counts⋅15 s^-1^⋅epoch^-1^), MVPA (≥287.5 counts⋅15 s^-1^⋅epoch^-1^), and TPA (≥25 counts⋅15 s^-1^⋅epoch^-1^) [46] will be summed using preschooler-specific cut-points, in *KineSoft* custom software (version 3.3.62; KineSoft, Loughborough, UK). To account for the varying times that participants spend in care, and to facilitate comparison with other studies, hourly rates and percentages of monitoring time of total physical activity will be calculated.

#### Secondary outcome measures

##### Preschoolers’ levels of sedentary time

Preschoolers’ sedentary time will also be objectively measured using Actical accelerometers.

##### Family demographics

Parents/guardians of participating preschoolers will complete a demographic questionnaire at baseline to gather information regarding potential correlates of this group’s physical activity levels, such as preschooler’s age, sex, ethnic origin, yearly family income, parent/guardian education levels, as well as the child’s level of physical activity participation outside of childcare (e.g., swimming lessons, soccer, etc.). This tool will also ask parents/guardians to report the number of minutes of MVPA in which they typically participate per week. Many of the variables collected in this questionnaire will be used as predictors of children’s physical activity levels and sedentary time in our analyses.

##### Preschoolers’ anthropometric measurements

We will measure the height (using a Seca 214 “Road Rod” Portable Stadiometer; nearest 0.1 cm), weight (using a Tanita 700-TBF300GS Body Fat Analyzer w/Goal Setter scale; nearest 0.1 kg), and waist circumference (using a measuring tape; nearest 0.1 cm) of participating preschoolers, at baseline, post-intervention, and at 6- and 12-month follow-up. Preschoolers will wear light clothing (e.g., sweaters and shoes will be removed) while measurements are conducted. All measurements will be taken twice, and where discrepancies are present, the average score will be used. Measurements will be conducted in a private area of the classroom or in the hallway to ensure participant comfort and privacy. The height and weight data collected will be used to calculate the child’s BMI percentile.

##### Quality of life

The Pediatric Quality of Life Inventory 4.0 (PedsQL) [47] is a reliable and valid inventory for the assessment of health-related quality of life within the preschool population (age 2-4 years). The PedsQL has been shown to have an internal consistency reliability of 0.90 for the total scale score for parent-proxy report. Moreover, this tool can accurately distinguish between healthy children and those with acute/chronic health conditions, and has demonstrated sensitivity to the severity of illness within the preschool population [48]. For this study, the parent report for children – PedsQL 4.0 Generic Core Scales will be used.

##### Child temperament

Previous research has recognized preschoolers’ temperament as possibly influencing their engagement in physical activity within childcare [42]. As such, the Children’s Behaviour Questionnaire (CBQ; very short form) [49, 50] will be administered. Used with children age 3 to 7 years old, this tool determines temperament across the dimensions of effortful control, surgency/extraversion, and affectivity (all via parent-report). Having exhibited acceptable internal consistency, criterion validity, longitudinal stability, and cross-informant agreement [49], this tool will be administered at all four data collection time points.

##### Outdoor play opportunities

For the duration of the intervention period (i.e., 8 weeks), childcare staff in both the intervention and control groups will record participating children’s daily outdoor play periods (and/or reasons why outdoor playtime was not possible for a particular day). This will be done to assess fidelity to the intervention parameters (i.e., 4 × 30 minutes of outdoor play for intervention group and 2 × 60 minutes for control group) and to maintain accountability.

##### Childcare providers’ physical activity and knowledge

The Childcare Provider Physical Activity Questionnaire, consisting of the validated short-form International Physical Activity Questionnaire (IPAQ-SF) [51], along with questions devoted to activity knowledge and practice (e.g., knowledge of physical activity and sedentary behaviour guidelines for the early years, description of centre policies for physical activity and screen-viewing [if any], etc.), will be administered to childcare providers (both control and intervention group) at the onset of the intervention to examine their current physical activity levels, knowledge, and practice within their childcare centres. Childcare providers have acknowledged their influence on preschoolers’ activity levels, as such, it is important to examine their own knowledge and practice [42]. Following baseline, this questionnaire will be re-administered immediately post-intervention and at 6- and 12-month follow-up to determine if any changes in their physical activity-related knowledge and behaviours occurred and whether they were sustained after the intervention.

##### Childcare providers’ physical activity-related self-efficacy

Childcare providers have noted lack of self-efficacy as a barrier to facilitating physical activity engagement in early learning environments [52]. The Childcare Provider Physical Activity Self-Efficacy questionnaire, developed for the purpose of the present study (based on Bandura’s *Guide for Constructing Self-Efficacy Scales*) [53], will be administered at baseline, post-intervention, 6- and 12-month follow-up to assess whether a significant change occurred in providers’ confidence to engage preschoolers in physical activity while in care (as well as whether this change was maintained after the intervention). Sample questions include: *How confident are you that you can engage the preschool childcare in your care in physical activity for 180 minutes each day?*, and *How confident are you that you can still engage the preschool childcare in your are in physical activity outdoors when the weather is poor/unfavourable?*

##### Childcare provider demographics

Childcare providers will be asked to complete a brief, 6-item demographic questionnaire (i.e., sex, age, ethnicity, highest levels of education, employment status [full-time/part-time], and years of experience). This information will be used to describe the sample of participating childcare providers.

##### Program evaluation

It is important to ensure that this program is both effective and feasible for staff to implement. As part of the process evaluation (to ensure we receive feedback from all childcare providers assigned to the experimental condition), questions regarding the feasibility and acceptability of the intervention will be asked post-intervention using a Program Evaluation Survey. This tool will be developed by the research team in consultation with childcare stakeholders, and will ask questions (on a 5 point Likert scale) about the feasibility, effectiveness, and enjoyment of the SPACE intervention along with open ended questions about challenges experienced with implementation.

Focus groups or interviews with childcare providers from the experimental condition will also be undertaken post-intervention to gather more in-depth perspectives regarding the appropriateness of the SPACE intervention, the feasibility of implementation, as well as suggestions for improvement. These qualitative discussions will provide rich data regarding the logistical challenges, and the strengths and weaknesses of the SPACE intervention that could not be captured on a questionnaire. An experienced moderator, using a semi-structured interview guide, will facilitate the discussions. Sample questions include: *What challenges did you experience when implementing the SPACE intervention?, What solutions did you undertake to deal with these challenges?,* and, *What characteristics of the SPACE intervention do you feel were most appropriate for increasing physical activity participation among preschoolers?* Focus groups and interviews will be audio recorded and transcribed verbatim.

### Analyses

Descriptive statistics and standard deviations will be calculated to describe the study participants and the participating childcare centres.

To evaluate the effectiveness of the introduced intervention on preschoolers’ physical activity levels (i.e., TPA), a 2 × 4 split-plot ANCOVA will be undertaken, in which 2 groups (experimental and control) are compared across 4 time points (baseline, post-intervention, 6- and 12-month follow-up), with child temperament and demographic information (e.g., age, sex) being entered as covariates. We will also examine the immediate post-intervention impact on TPA, using a 2 × 2 split-plot ANCOVA. Independent sample *t*-tests will be completed to explore sex differences in activity levels at the various data collection time points. Since a random cluster sampling strategy will be used, all quantitative analyses will include “childcare centre” as a random factor to account for clustering effect. For all repeated measures analyses, the effects of the independent variables on these outcomes will be evaluated at both a multivariate and univariate level, with appropriate post-hoc testing performed on all significant omnibus effects.

To evaluate changes in participants’ sedentary activity, BMI percentiles, and quality of life, a 2 × 4 split-plot MANCOVA will be undertaken, in which 2 groups (experimental and control) will be compared across 4 time points (baseline, post-intervention, 6-, and 12-month follow-up). Sex differences will be explored using independent sample *t*-tests. To analyze changes in childcare providers’ physical activity knowledge and self-efficacy, a 2 × 4 split-plot MANOVA (2 groups × 4 time points) will be completed to examine if changes are evident after the intervention. Means and standard deviations will be calculated to gather childcare providers’ consensus regarding the feasibility, applicability, and usability of the SPACE intervention (collected from the *Program Evaluation Survey*).

To analyze the qualitative focus groups data, inductive content analysis will be completed independently by two researchers to explore emerging themes [54]. All analyses will be conducted in QSR NVivo software. In line with Guba and Lincoln’s recommendations, steps will be taken to ensure data trustworthiness (e.g., credibility, transferability, dependability, and confirmability) [55].

## Discussion

The low levels of physical activity and high rates of sedentary time among the preschool population are problematic [1, 2, 14]. In the interest of creating an evidence-informed physical activity intervention, the SPACE study was developed following the PRECEDE-PROCEED model for health promotion program planning [30]. Our research team undertook a needs assessment with childcare staff [56] to understand the challenges of implementing physical activity in their facilities, followed by a meta-analysis to examine the literature and understand the characteristics of interventions that have been previously successful with this population [24]. Based on these two sources of information, the SPACE intervention was developed with the primary aim of increasing physical activity among preschoolers enrolled in centre-based childcare. While other interventions have been implemented with this cohort elsewhere [28, 29], this is the first study, to our knowledge, to tackle shorter, more frequent bouts of *outdoor* playtime, combined with environmental modifications and staff training. Moreover, this is only the second childcare intervention undertaken in Canada – the first intervention by Adamo and colleagues adopted a different approach which focused on staff training and offered a resource manual (that included weekly schedules) to facilitate staff-led physical activity time [29]. Given the young ages of preschoolers, as well as their dependence on the adults in their lives, childcare staff are in a key position to encourage physical activity among this group. Interventions such as these are important given that childcare staff [56], as well as early childhood education students [57], have acknowledged their desire for additional physical activity training. Modifying the frequency of outdoor playtime in childcare facilities represents a relatively easy, cost-effective, and therefore sustainable, change for childcare centres. Given the link between outdoor playtime and increased physical activity noted among the preschool population [27], this approach was reasonable and evidence-informed. By establishing healthy active behaviours during the early years, preschoolers may be more likely to continue these behaviours long-term.

Although we anticipate that the SPACE study will be effective at increasing TPA levels among preschoolers, it is also important that we gather information about the appropriateness of this intervention from front-line staff members. While the study was designed to transpire during spring/summer months, in an effort to reduce seasonal effects on physical activity [58] and the challenge of getting preschoolers outdoors four times a day in cold-weather clothing, other challenges or practicality issues may be present. Given the success of this intervention lies on the buy-in and participation of childcare staff, it is imperative that we gather this information from frontline educators.

The SPACE study represents a novel, evidence-informed approach to changing physical activity behaviours among this young cohort. It is important that we share the intervention components and evaluation plans with researchers and childcare stakeholders who may be interested in implementing a similar intervention. The results of the SPACE study will be disseminated to early year’s policy makers, childcare staff and directors, and parents/guardians to ensure those responsible for the guidelines and practices within these facilities have the knowledge necessary to make decisions in support of healthy active behaviours among preschoolers.

## Abbreviations

CONSORT: Consolidated Standards of Reporting Trials
ECE: early childhood education
IPAQ-SF: International Physical Activity Questionnaire – short form
ISRCT: International Standard Randomised Controlled Trial Number
LPA: light physical activity
MVPA: moderate-to-vigorous physical activity
REB: research ethics board
SPACE: Supporting Physical Activity in the Childcare Environment
TPA: total physical activity
